# Anti-tumor therapy with macroencapsulated endostatin producer cells

**DOI:** 10.1186/1472-6750-10-19

**Published:** 2010-03-02

**Authors:** Danielle B Rodrigues, Roger Chammas, Natália V Malavasi, Patrícia LN da Costa, Rosa M Chura-Chambi, Keli N Balduino, Ligia Morganti

**Affiliations:** 1 Centro de Biotecnologia, Instituto de Pesquisas Energéticas e Nucleares (IPEN), São Paulo, SP, Brazil; 2 Laboratório de Oncologia Experimental, Faculdade de Medicina da Universidade de São Paulo (FM-USP), São Paulo, SP, Brazil

## Abstract

**Background:**

Theracyte is a polytetrafluoroethylene membrane macroencapsulation system designed to induce neovascularization at the tissue interface, protecting the cells from host's immune rejection, thereby circumventing the problem of limited half-life and variation in circulating levels. Endostatin is a potent inhibitor of angiogenesis and tumor growth. Continuous delivery of endostatin improves the efficacy and potency of the antitumoral therapy. The purpose of this study was to determine whether recombinant fibroblasts expressing endostatin encapsulated in Theracyte immunoisolation devices can be used for delivery of this therapeutic protein for treatment of mice bearing B16F10 melanoma and Ehrlich tumors.

**Results:**

Mice were inoculated subcutaneously with melanoma (B16F10 cells) or Ehrlich tumor cells at the foot pads. Treatment began when tumor thickness had reached 0.5 mm, by subcutaneous implantation of 10^7 ^recombinant encapsulated or non-encapsulated endostatin producer cells. Similar melanoma growth inhibition was obtained for mice treated with encapsulated or non-encapsulated endostatin-expressing cells. The treatment of mice bearing melanoma tumor with encapsulated endostatin-expressing cells was decreased by 50.0%, whereas a decrease of 56.7% in tumor thickness was obtained for mice treated with non-encapsulated cells. Treatment of Ehrlich tumor-bearing mice with non-encapsulated endostatin-expressing cells reduced tumor thickness by 52.4%, whereas lower tumor growth inhibition was obtained for mice treated with encapsulated endostatin-expressing cells: 24.2%. Encapsulated endostatin-secreting fibroblasts failed to survive until the end of the treatment. However, endostatin release from the devices to the surrounding tissues was confirmed by immunostaining. Decrease in vascular structures, functional vessels and extension of the vascular area were observed in melanoma microenvironments.

**Conclusions:**

This study indicates that immunoisolation devices containing endostatin-expressing cells are effective for the inhibition of the growth of melanoma and Ehrlich tumors.

Macroencapsulation of engineered cells is therefore a reliable platform for the refinement of innovative therapeutic strategies against tumors.

## Background

Angiogenesis, the formation of new blood vessels from existing capillaries, is required for tumors to expand beyond 1-2 mm^3 ^in size. It is also essential for the growth and persistence of solid tumors and their metastases [1-3]. Endostatin is a specific angiogenesis inhibitor that prevents vascular endothelial cells from proliferating and migrating in response to proangiogenic proteins. This inhibitor can potently prevent tumor growth without inducing toxicity or acquired drug resistance [4,5]. However, injection of endostatin leads to its rapid clearing from the circulation [6].

Encapsulation of recombinant cells expressing therapeutic proteins within semi-permeable devices for *in vivo *protein delivery to systemic circulation is a potentially sustainable, long-term release system. The semi-permeable membrane allows for the exchange of nutrients and oxygen between the implanted cells and the host, besides protecting the cells from rejection by the immune system. This system can circumvent the problems of limited half-lives and variation in circulating levels of therapeutically active proteins. There are reports on the treatment of solid tumors using the local delivery of endostatin by either implantation of alginate microcapsules containing genetically engineered cells that can produce this protein in the vicinity of the tumor site [6,7] or transplantation of these devices into the peritoneal cavities of model animals [8-10]. However, there is no possibility of withdrawing the alginate microcapsules containing the genetically engineered cells in case of undesired side effects. Another likely problem is the possibility of a rupture of the microcapsules and liberation of the genetically engineered cells, which leads to attack from the host's immune system.

The Theracyte™ immunoisolation device is a polytetrafluoroethylene (PTFE) membrane macroencapsulation system comprised of a 0.4-μm pore cell-impermeable membrane, laminated to a 5-mm pore membrane. The microarchitecture of this membrane is designed to induce neovascularization at the tissue interface, and it can also protect the cells from host's immune rejection, thereby circumventing the problem of limited half-life. This membrane has been developed for implantation of cells capable of producing therapeutic proteins [11]. Besides, this device allows for high density packing of encapsulated cells in a relatively small area, and it has been found to be allo-protective [12]. The Theracyte device can be easily removed altogether with the cells in the case of undesired side effects or at the end of the treatment.

In the present study, mice fibroblasts (LM Murine fibroblast cells) engineered for continuous endostatin secretion were encapsulated in Theracyte™ immunoisolation devices subcutaneously implanted in mice at a site distant from the tumors. Aiming at optimizing the antitumor treatment, two protocols were tested for implantation of the cells within the membranes. In the first protocol, the devices were pre-implanted in the animals and, after wound healing and tumor growth to a thickness of 0.5 mm (approximately 14 days), the endostatin producer cells were injected inside these devices. In the second protocol, the endostatin producer cells were injected into the devices, which were then implanted in the mice that already presented a tumor. The ability of the non-encapsulated and encapsulated cells to secrete biologically active endostatin capable of inhibiting tumor growth was evaluated.

## Results

### Formation of blood vessels occurs in the stroma in the vicinity of the devices

An important factor related to the survival of recombinant cells is the proper delivery of oxygen and nutrients to the cells inside the macroimmunoisolation devices. Theracyte devices are designed to induce the formation of vascular structures in its vicinity.

**Figure 1:**
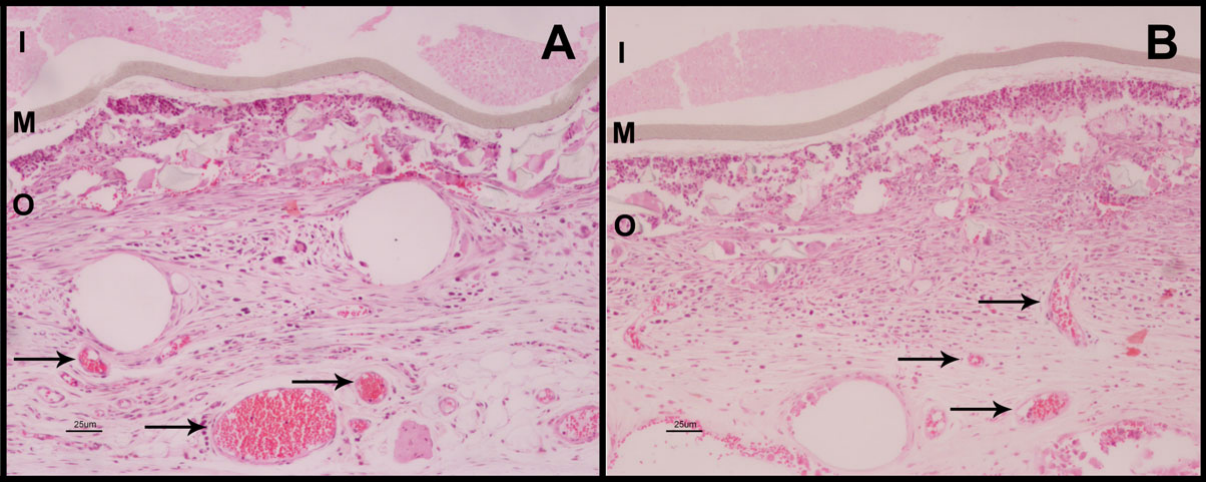
**The tissue adjacent to the devices was vascularized but cells inside the devices are not viable**. **(A **and **B)**- Histological analyses (stained with H.E.) of the environment around the device. Arrows show new blood vessels, which reveals that it is biologically inert and promotes neovascularization, and that the cells are inside the device. No viable endostatin producer cells were noted; **(A) **Histological analyses of the environment around the device which was pre-implanted;**(B) **Histological analyses of the environment around the device which was implanted concomitantly with the cells.

One way to circumvent the possible negative effect of endostatin secreted by the cells inside the device on its own, via reduced vascularization around the devices, was to pre-implant empty immunoisolation devices in the animals, followed by induction of the Ehrlich or melanoma tumors after an adequate time. Treatment was initiated when the tumor thickness had reached 0.5 mm, by injecting cells expressing endostatin into the pre-implanted devices, approximately 14 days after device implantation. In order to verify whether the pre-implantation of the devices is necessary, implantation of devices already containing endostatin producer cells was also performed for melanoma tumors, despite the possible deleterious effect of endostatin on the neovascularization of the wounds. To this end, treatment began by implanting Theracyte devices already containing the endostatin-expressing cells after the melanoma had grown to 0.5 mm thickness. Withdrawal of the devices was performed on the 14^th ^treatment day. The presence of blood vessels, indicative of neovascularization, was observed in the stroma in the vicinity of the immunoisolation system for both protocols of implantation of the H.E.-stained slices (Figures 1A and 1B). However, apparently no viable cells could be detected in the interior of the devices. Adhesion of LM cells to the internal face of the device membrane was seen, since this is probably the area with larger readiness of oxygen and nutrients.

### Tumor growth is reduced by treatment with endostatin-expressing cells

The treatment of mice bearing Ehrlich or melanoma tumors with encapsulated or non-encapsulated cells began when tumor thicknesses at the pads had reached 0.5 mm.

**Figure 2:**
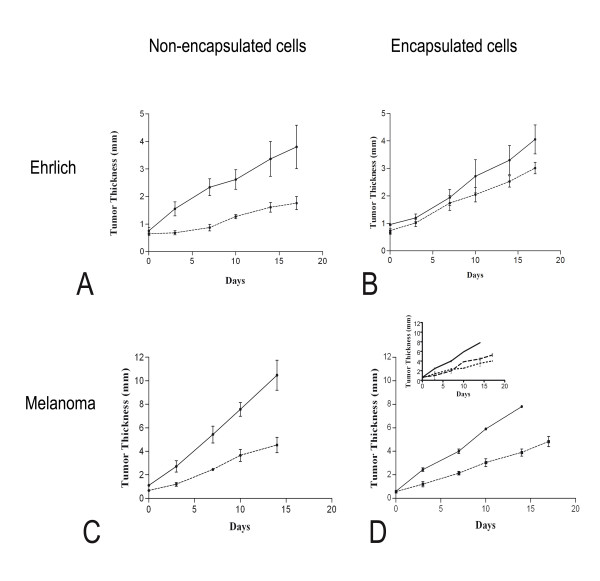
**Endostatin delivered by engineered cells impaired tumor growth**. **(A), **Solid lines: control group treated with LM(pSecTag) non-encapsulated cells (n = 6); Dashed lines: group treated with LM(pSecTag-end) non-encapsulated cells (n = 4); (**B), **Solid line: control group treated with LM(pSecTag) encapsulated cells injected into pre-implanted devices (n = 3); Dashed line: group treated with LM(pSecTag-end) injected into pre-implanted devices (n = 5); **(C)**, Solid lines: control group treated with LM(pSecTag) non-encapsulated cells (n = 4); Dashed lines: group treated with LM(pSecTag-end) non-encapsulated cells (n = 4); (**D), **Solid line: control group treated with LM(pSecTag) encapsulated cells injected into devices which were then implanted into mice (n = 2); Dashed lines: group treated with LM(pSecTag-end) encapsulated cells using both protocols of implantation (n = 5); **Inset D **shows the same data as in D, Solid line: control group treated with LM(pSecTag) encapsulated cells injected into devices which were then implanted into mice (n = 2); Dotted line: group treated with LM(pSecTag-end) encapsulated cells injected into devices which were then implanted into mice (n = 3); Dashed line: group treated with 10^7 ^LM(pSecTag-end) encapsulated cells, injected into the pre-implanted devices (n = 2).

Ehrlich tumor growth inhibition was observed for mice treated with LM(pSecTag-end) non-encapsulated cells. The average of Ehrlich tumor thickness at the mice pads was 1.28 ± 0.28 mm and 2.69 ± 1.13 mm for the treated and mock-treated groups of non-encapsulated cells, respectively, at day 17 (Figure 2A). However, tumors were observed where LM(pSecTag-end) or LM(pSecTag) cells were injected. A lower tumor growth inhibition was obtained for Ehrlich tumor-bearing mice treated with encapsulated endostatin producer cells. An average tumor thickness of 2.00 ± 0.37 mm and 2.64 ± 0.74 mm were obtained at day 17 for the animals treated with encapsulated LM(pSecTag-end) cells and encapsulated LM(pSecTag) cells, respectively (Figure 2B).

Figure 2C shows the average melanoma tumor thickness at the pads of animals treated with non-encapsulated endostatin-expressing cells. The average thickness of the tumors in the mice treated with the non-encapsulated LM(pSecTag-end) cells was 4.53 ± 1.31 mm, whereas the average tumor thickness in the control group, treated with the LM(pSecTag) cells, was 10.47 ± 2.54 mm, a growth reduction of 54.7%. Tumor growth inhibition was also obtained for the mice treated with encapsulated LM(pSecTag-end) using both protocols of Theracyte implantation (Figure 2D). The differences between tumor growth reduction for mice treated with pre-implanted devices and devices implanted with the cells were small, although the number of animals was low, two and three, respectively. The average thickness of the melanoma tumor of mice treated via injection of cells into pre-implanted devices was 4.45 ± 0.07 mm and 7.80 ± 0.14 mm for the treated animals and control group, respectively. The values of melanoma tumor thickness obtained in the group treated with devices implanted simultaneously with the endostatin-producer cells was 3.55 ± 0.64 mm at day 14.

### Endostatin induces inhibition of angiogenesis in the melanoma tumors

**Figure 3:**
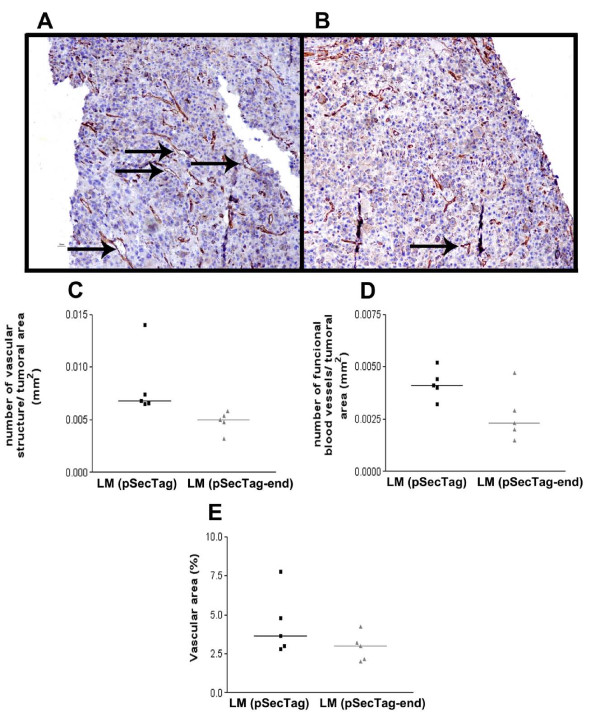
**Neovascularization on melanoma tumor was impaired by endostatin**. (A and B) - Immunohistochemical analyses of the melanoma tumor microenvironment, after 7 days of treatment, showing the differences found in the vascular structure in the (A) control mice treated with LM(pSecTag) cells and (B) mice treated with LM(pSecTag-end) cells. The neovascularization was quantitatively analyzed in the tumor tissue immunohistochemistry stained using the CD34 antibody (C) Number of vascular structures (n = 5) (p = 0.008) ; (D) Number of functional vasculature (vessel with erythrocyte inside) (n = 5) (p = 0.047); and (E) vascular area (n = 5) (p = 0.2). Mean +/- SD.

Analysis of the B16-F10 melanoma tumors after 7 days of treatment using 10^7 ^non-encapsulated cells was performed, in order to evaluate the efficacy of endostatin on angiogenesis. The microvascular density was evaluated using the CD34 antibody. Quantitative analysis of the sections showed a decrease of 41.5% in the number of vascular structures, 35.9% in the number of functional vessels, and 33.5% in the extension of the vascular area compared with the tumors of animals treated with mock cells (Figure 3). The solid Ehrlich tumor was shown to be poorly vascularized (data not shown).

### Endostatin secreted by the cells inside the devices reaches the adjacent stroma

**Figure 4:**
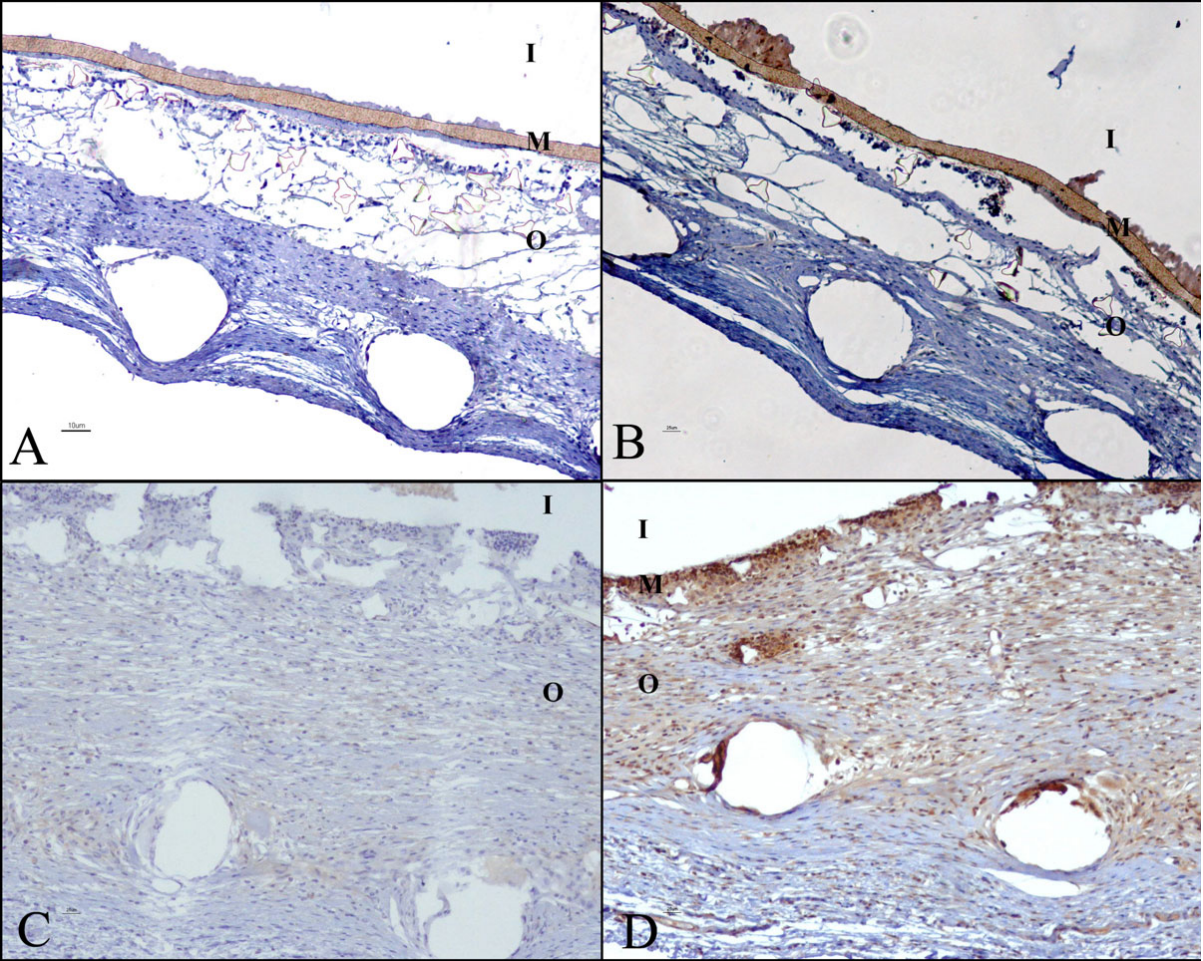
**Endostatin is present in the stroma adjacent to the device**. **(A and B) **Immunohistochemical analyses using BSA in the tissue around the devices with **(A) **LM(pSecTag) cells; **(B) **LM (pSecTag-end) cells, showing the absence of reaction in the stroma adjacent to the devices; **(C **and **D)- **Immunohistochemical analyses using endostatin antibody of the environment around the device; **(C) **Immunohistochemical analyses from mice with melanoma tumor treated with encapsulated LM(pSecTag) cells, sustaining that there is no endostatin in the tissues adjacent to the device; **(D) **Immunohistochemical analyses from mice with melanoma tumor treated with encapsulated LM(pSecTag-end) cells, indicating the presence of endostatin in the stroma adjacent to the device which was implanted with endostatin-expressing cells. The reaction that shows up in the cells inside the device is due to an unspecific binding. The reaction in the adjacent tissue is due to the presence of endostatin . **(i)- **region inside the device; **(m)- **membrane; **(o)- **region outside the device.

Immunohistochemistry with endostatin antibody revealed a gradual dispersion of this protein in the tissue around the device (Figure 4), which was withdrawn from melanoma-bearing mice on the 14^th ^treatment day. This result indicates that endostatin secreted by the confined recombinant cells permeated through the membrane, outstretching to the surrounding tissues.

## Discussion

We describe a therapeutic protocol for the antiangiogenic treatment of the Ehrlich and melanoma tumors induced in C3H-HePas and C57Bl/6 mice, respectively. The treatment consists in using endostatin-expressing murine fibroblasts encapsulated in macroimmunoisolation devices (Theracyte™). Immunoisolation at sufficiently high cell densities for practical therapy might be enabled by membranes that facilitate neovascularization at the membrane of the device-tissue interface. This is important for the sustained supply of oxygen and nutrients to the cells inside the devices as well as for the delivery of therapeutic proteins to the surrounding tissues [11]. Low densities of cells inside macroimmunoisolation devices can be achieved because of mean oxygen tension resulting from poor vascularization in the vicinity of the implant. Theracyte devices display 5 μm PTFE pores that stimulate formation of vascular structures via penetration of host cells. This leads to secretion of angiogenic factors and/or factors by the host cells, which overcome an existing inhibition of neovascularization, laminated to smaller pore (0.4 μm) inner membrane, thereby preventing cell entry into the immunoisolation devices. In our study, examination of the local response to the implanted cells shows the presence of blood vessels in the vicinity of the devices, ratifying the supposition that the Theracyte™ membrane induces neovascularization in the tissue adjacent to it. Our data indicate that, after 14 days, the encapsulated cells used for the treatment are apparently nonviable. It is probable that, despite the existence of neovascularization, the supply of nutrients and oxygen is not sufficient to keep transplanted cells alive for a long time. Nevertheless, the subcutaneous growth of melanoma and Ehrlich tumors was inhibited with both protocols used for implantation of immunoisolated endostatin-secreting cells. The consistent effect observed in tumor growth is likely attributable to the elevation of the serum levels of endostatin. In the first treatment protocol, the device was pre-implanted and, after wound healing and neovascularization in the vicinity of the device, the endostatin-expressing LM(pSecTag-end) cells were inoculated into the capsule. Implantation of the device before the injection of the LM(pSecTag-end) cells aimed at improving vascularization at the host tissue/device membrane interface before implantation of the endostatin-secreting cells, which would possibly interfere negatively with blood vessel formation, cell viability, and thus response to the treatment. A 42.4% reduction in melanoma growth was obtained. Endostatin acts preferentially in tumor neovascularization [13,14], and the most evident effect of endostatin on the vascularization of wounds in non-tumoral woven is the formation of a larger number of nonfunctional vessels. In the second protocol of tumor treatment, devices already containing the endostatin-producer cells were implanted, and a slightly larger tumor reduction was obtained for tumor growth: 54.5% at the 14^th ^treatment day. This result indicates that either the effect of endostatin was not as significant for neovascularization of normal tissue as it was effective for the treatment of the tumors, or that there are other factors implicated in the survival of the cells inside the devices. These factors can possibly supplant the negative effect of endostatin on the formation of new blood vessels.

Different approaches will be necessary to sustain endostatin serum levels through larger periods. Replenishing macrocapsules with endostatin producing cells is among the possible strategies. Despite the limited number of animals tested in this study, we could show that filling implanted capsules is possible and yields similar results as compared with experiments using filled capsules. Alternatively, new devices can be implanted. Implantation of the immunoisolation devices already containing the endostatin-producing cells is convenient because the beginning of the treatment is not delayed by pre-implantation of the empty device, not to mention that only one surgery is necessary in this case.

A protocol for endostatin delivery to the systemic circulation of mice using recombinant CHO cells, expressing high endostatin levels, encapsulated in Theracyte™ bioisolating devices was described. Throughout the two-month study, constant and high endostatin levels of up to 3.7 μg/ml were detected in the plasma of the mice implanted with the devices [15]. The use of CHO engineered cells, which apparently are more resistant than LM to the hypoxic microenvironment of the immunoisolation devices is other alternative to keep endostatin serum levels.

The melanoma is a highly vascularized tumor, whereas the solid form of the Ehrlich tumor presents much lower vascularization rates. The more vascularized the tumor, the higher the probability of obtaining tumor regression by treatment with angiogenesis suppression factors [16-18]. It has already been suggested that, besides the classical antiangiogenic effect of endostatin, a second mechanism may also be implied in endostatin-dependent tumor regression, associated with tumor infiltration of leucocytes promoted by leukocyte-endothelium interactions, indicating that a combination of both these effects leads to tumor regression [19,20]. Histology of Ehrlich tumors showed that besides necrosis, tumors of the treated group also displayed more leukocyte infiltration (data not shown). More studies should be conducted to verify this relationship.

The effect of endostatin produced by LM(pSecTag-end) cells on melanoma neovascularization in mice was determined by analysis of the vascular structures. A smaller number of vascular structures were obtained in the mice treated with the endostatin-expressing cells compared with the control mice. The number of functional vessels in the tumor area was also smaller in the treated mice. In order to confirm "in vivo" endostatin expression by the cells within the devices, imunohistochemistry was accomplished. The presence of endostatin in the tissues adjacent to the device was demonstrated, showing production, liberation and diffusion of this protein to the stroma in the vicinity of the device. These results endorse the hypothesis that tumor regression was obtained by action of the endostatin released by the immunoisolated cells expressing this protein.

Regarding the comparison between treatment using non-encapsulated cells or cells wrapped up in alginate microcapsules [6,7,9,10], the advantages of the use of macroencapsulated cells are: (1) the possibility of removing the devices in the case of adverse reactions or at the end of the treatment; (2) escape of potentially tumorigenic endostatin producing cells from the macrocapsules is virtually impossible; (3) the system described herein allows the use of universal allogeneic endostatin producer cells, avoiding the host's immune rejection.

Studies using the Theracyte™ system were performed mainly by implantating islets for diabetes treatment [21-25]. Brauker and cols [12]. employed an immunoisolation device with a bi-laminated PTFE membrane, similar to the one used in the Theracyte™ system, and demonstrated that immunoisolated allograft or isographs survived for one year, while xenografts were destroyed within 3 weeks. This was explained by possible xenogenic tissue killing through the local accumulation of inflammatory cells, mediated by liberation of antigens from the Theracyte immunoisolation device and presentation by an indirect pathway. High viability of recombinant isogenic or allogenic cells expressing erythropoietin in Theracyte™ devices one year after implantation of the capsule without evidences of immune response [26] was also described. In the present work we used isogenic cells (LM (tk^-^)) for the treatment of C3H-HePas mice, while allogenic ones were employed to treat C57Bl/6 mice. The small viability time of the cells inside the immunoisolation devices is not associated with host rejection, because this fact was observed even when isogenic cells were implanted. Also, cells of the immune system were not observed in the vicinity of the device. A possibility for future studies is the implantation of a new device containing LM(pSecTag-end) cells after some time. Alternatively, the periodic injection of endostatin-expressing cells within the devices could be investigated.

## Conclusions

This is the first demonstration of the use of macroencapsulated engineered cells for delivery of anti-angiogenic factors, such as endostatin, in tumor treatment. The data demonstrate that, despite the low viability of encapsulated cells at the end of the treatment, genetically engineered encapsulated cells provide a viable vehicle for the delivery of endostatin for antiangiogenic treatment of tumors.

## Methods

### Cells and Culture

B16-F10 cells, belonging to a highly malignant melanoma cell line derived from a tumor that arose spontaneously in a C57BL/6 mouse, were obtained from ATCC (CRL-6475™, Manassas, VA, USA) and cultured with Dulbecco's modified Eagle's medium DMEM (Cultilab, Campinas, Brazil) supplemented with 10% heat-inactivated fetal bovine serum (Gibco, Invitrogen, Grand Island, NY, USA), 100 U/mL penicillin (Gibco, Invitrogen, Grand Island, NY, USA), 50 mg/mL streptomycin (Gibco, Invitrogen, Grand Island, NY, USA), 2 mM L-glutamine (Gibco, Invitrogen, Grand Island, NY, USA), 2.5 μg/mL amphotericin B, (Gibco, Invitrogen, Grand Island, NY, USA), 2.2 g/L sodium bicarbonate (Gibco-BRL, Gathersburg, USA), and 110 mg/L sodium piruvate (Gibco, Invitrogen, Grand Island, NY, USA). Cells were routinely cultured in 250 mL T-flasks and incubated at 37°C in a humidified 5% CO_2 _atmosphere. For melanoma development, C57Bl/6 mice were injected s.c. with 10^6 ^B16-F10 murine melanoma cells in the pads of their feet. Palpable tumors appeared within 10-15 days after injection.

Ehrlich tumor cells were maintained in ascite in C3H(HePAS) mice. For the development of solid tumors, the ascitic liquid was withdrawn, the cells were washed in sterile PBS, and 4 × 10^6 ^tumor cell were injected s.c. in the pads of the C3H mice feet.

LM Murine Fibroblasts (ATCC n°: CCL-1.3™, Manassas, VA, USA), transfected with either the empty pSecTag vector (Invitrogen, Carlsbad, CA, USA) or the pSecTag-end that contains the murine endostatin gene (ATCC, n. 63404, Manassas, VA, EUA), were selected with 600 μg/mL hygromycin (Invitrogen, Carlsbad, E.U.A) for 14 days. The LM(pSecTag-end) cells presented an expression of 0.4 μg murine endostatin/10^6 ^cells/24 hours and were maintained in the same conditions as those described for the B16-F10 cells.

### Animals

The animal experiments were approved by the IPEN ethics committee for animal research (CEPA - protocol number: 18). Male C57BL/6 and C3H-HePas mice aged 6-10 weeks were used. They were acclimatized, caged in groups of up to five animals in a barrier care facility, and fed with animal chow and water *ad libitum*. C57Bl/6 and C3H mice were subcutaneously injected in the pads of their mice feet with 10^6 ^B16-F10 murine melanoma cells or 4 × 10^6 ^Ehrlich cells, respectively, in a volume of 0.2 mL sterile phosphate buffered saline solution (PBS). Mice feet were measured every two or three days with an electronic caliper (YT203, Digimes Yato Pro-Cal, Guangdong, China), and the tumor thickness was obtained by calculating the differences between the measures of the tumor-bearing and the normal feet.

### Tumor treatment using endostatin producer cells

Three distinct protocols to evaluate tumor treatment with endostatin producer cells were employed. In the first one, 10^7 ^LM(pSecTag-end) cells, containing the murine endostatin gene, were "freely" injected into the ventral region in a volume of 20 μL DMEM medium after melanoma or Ehrlich tumor growth to 0.5 mm thickness. In the second protocol, performed for melanoma or Ehrlich tumor-bearing mice, the Theracyte™ immunoisolation capsules were pre-implanted s.c. in the back of the animals and, after wound healing and tumor growth to 0.5 mm thickness, 10^7 ^LM(pSecTag-end) cells were injected into the immunoisolation device. A third procedure, performed only for melanoma-bearing mice, involved implantation of the immunoisolation capsule already containing 10^7 ^LM(pSecTag-end) cells in the animals, after tumor thickness had reached 0.5 mm.

### Loading endostatin expressing cells into a Theracyte™ capsule and its implantation in the animals

A Theracyte™ capsule (Baxter Healthcare, Deerfield, IL) with a nominal internal volume of 20 μL within a polytetrafluoroethylene (PTFE), bilayer membrane wall was employed. Theracyte™ capsules were sterilized using 100%, 70% ethanol and sterile normal saline washes. Mice were anaesthetized with a mixture of ketamine (65 mg/kg) and xylazine (5 mg/Kg), and the dorsal area was swabbed with betadine. As mentioned in the former section, two implantation procedures were adopted. In the first procedure, a dorsal midline incision was made in the animal skin, a pocket was created by blunt dissection with a hemostat, and the devices (with the port entrance sealed by means of a silicon adhesive) were inserted into the back of the animal. The incision was then closed by surgical suture. Another surgery was carried out after wound healing, tumor growth and suitable neovascularization around the device (approximately 14 days later), so that a suspension of 10^7 ^cells expressing endostatin (treated animals) or a suspension containing 10^7 ^LM(pSecTag) cells (mock-treated animals) was injected into the pre-implanted device with the aid of a Hamilton syringe. In the second implantation procedure, tumor cells were injected into the animal prior to capsule implantation. When tumor thickness had reached approximately 0.5 mm, 10^7 ^cells were injected into the immunoisolation devices, which were then sealed and implanted in the subcutaneous site of anesthetized animals.

### Histology and immunohistochemistry analysis

Animals were killed at the end of the experiment. The devices were excised, washed in PBS, fixed in PBS-buffered 3.7% formalin for 24 h, and processed according to routine histopathological protocols. The histological analysis was performed in 4-μm sections stained with hematoxylin and eosin. For the immunohistochemical detection of endostatin, sections of the devices were processed and subjected to the antigen retrieval method using anti-endostatin antibody (Anti - Rabbit AB1880, Chemicon, EUA), followed by development with a diaminobenzidine-based detection system (SuperPicture Poly HRP conjugated, Zymed, CA), according to routine histopathological protocols.

For the angiogenic analysis, the animals treated with non-encapsulated LM cells were euthanized after one week of treatment. Tumors were excised, fixed, and embedded. The microvascular density was measured using the anti-CD34 antibody (Santa Cruz Biotechnology Inc., Santa Cruz, CA, USA) and quantified by comparing the number of grid intersections overlaying the vascular structures, the functional vascular structure, or the vascular area with the tumor region of the fields embodying the tumor area of each animal. In all the cases, the images were collected using the Digital Camera DXM1200F, Nikon.

## Authors' contributions

DBR carried out the experiments of treatment of the tumor-bearing animals, carried out the quantification of tumor vessels and helped to draft the manuscript; RC participated on the design of the study and gave final approval of the version to be published; NMV carried out the Elisa assays for endostatin quantification and helped on the *in vivo *assay; PLNC carried out the immunohistochemistry and helped on the experiments of quantification of tumor vessels; RMCC helped on the cell cultivation and on the *in vivo *assay; KNB helped on the in vivo assay; LM Conceived the study, participated in its design and coordination and helped to draft the manuscript. All authors read and approved the final manuscript.
